# *CD38* genetic variation is associated with increased personal distress to an emotional stimulus

**DOI:** 10.1038/s41598-024-53081-5

**Published:** 2024-01-31

**Authors:** Tanya L. Procyshyn, Laury-Ann Leclerc Bédard, Bernard J. Crespi, Jennifer A. Bartz

**Affiliations:** 1 Department of Psychiatry, University of Cambridge, Cambridge, CB2 8AH UK; 2 Department of Biological Sciences, Simon Fraser University, 8888 University Drive, Burnaby, V5A 1S6 Canada; 3 Department of Psychology, McGill University, 2001 McGill College Avenue, Montreal, H3A 1G1 Canada

**Keywords:** Behavioural genetics, Neuroscience, Psychology

## Abstract

Genetic variation in *CD38*—a putative oxytocin pathway gene—has been linked to higher oxytocin levels, empathy, and sensitive parenting, but also to more negative interpersonal outcomes (e.g., alienation from friends and family, poorer romantic relationship quality). To reconcile these seemingly contradictory findings, we drew upon the idea that *CD38* variation may heighten social-emotional sensitivity and, consequently, make individuals prone to negative emotions in distressing interpersonal situations. To test this hypothesis, we performed a secondary analysis of a dataset including participants’ (n = 171; 94 females) empathic concern (“sympathetic”) and distress-related (“anxious”) responses to an emotional video. Distress responses were higher for the CD38 rs3796863 AA/AC group vs. the CC group (p = 0.03, η^2^ = 0.027); however, there was no significant effect of genotype for empathic concern responses to the video or for indices of trait empathy. These findings provide preliminary evidence that, in the face of an interpersonal stressor, *CD38* genetic variation may predict more self-focused, aversive emotional reactions. More broadly, this finding highlights the need to adopt a more nuanced perspective in which the influence of oxytocin system variation (assessed by oxytocin-related genetic variation) should be considered in light of the social context.

## Introduction

The ability to form positive relationships with others has undeniable effects on our mental and physical health^1,2^. The neurohormone oxytocin plays a crucial role in social bonding across various contexts, including parent-infant bonding, romantic relationships, and group dynamics^3–6^. Variation in the human oxytocin system—which is commonly indexed using oxytocin-related genetic variants as a proxy—has thus attracted interest as a predictor of socially relevant outcomes. This line of research has shown that oxytocin-related genetic variants are associated with measures of empathy^7^, changes in default mode network connectivity following trauma^8^, brain activation during an emotion recognition task^9^, and likelihood of autism spectrum conditions^10^.

One oxytocin-related genetic variant studied in the context of social behaviour is the cluster of differentiation 38 (*CD38*) gene on human chromosome 4, which encodes a glycoprotein highly expressed in the brain that is involved in secretion of hypothalamic hormones like oxytocin^11,12^. Most studies of this gene have focused on the *CD38* single nucleotide polymorphism (SNP) rs3796863, for which the C variant is associated with higher likelihood of autism^13^. In a study of parents, Feldman et al.^14^ found that those with the “low risk” A allele (vs. the CC group) had higher plasma oxytocin levels and a more sensitive parenting style as measured by touch and gaze synchrony while interacting with their children. Similarly, in a large study of students in China, A allele carriers (vs. CC) reported stronger empathic responses to a fictional teacher with a medical condition and voluntarily donated more of their research-related earnings to the hypothetical teacher’s medical expenses^15^. Supported by evidence that *CD38* is crucial for oxytocin release and social behavior in rodents^16^, the findings of these two studies were interpreted to imply that the rs3796863 A allele is associated with higher oxytocin levels and more pro-social patterns of interpersonal interactions in humans.

Subsequent studies, however, have shown that the rs3796863 A allele is not always associated with positive or preferential social outcomes. McQuaid et al.^17^ found that university students with the AA genotype reported higher levels of alienation from parents and peers, suicidal ideation, and depressive symptoms. McInnis et al.^18^ reported similar patterns, such that university students with the AA genotype reported more unsupportive interactions with their peers, which was associated with greater negative mood. In a study of older adults, Tabak et al.^19^ reported a gene × environment interaction by which A allele carriers who experienced greater interpersonal stress over a period of six years had more negative outcomes in terms of social anxiety and, to a lesser extent, depression.

In the literature on romantic relationships, it is again the A allele that is associated with worse interpersonal outcomes. For example, Algoe and Way^20^ found that individuals with one or two A alleles (vs. CC) showed lower levels of gratitude towards their romantic partner—an experience that is thought to promote bonding—both in a laboratory-based interaction and in daily life. Similarly, Sadikaj et al.^21^ found that individuals with an A allele (vs. CC) displayed less communal behavior (e.g., expressing affection, smiling, etc.) during interactions with their romantic partner (based both on self- and partner-report); they also reported more negative affect, greater insecurity during these partner interactions, and lower levels of overall relationship quality. Lastly, in a study of newlywed couples, Makhanova et al.^22^ found that A allele carriers (vs. CC) had steeper declines in relationship satisfaction during their first three years of marriage.

These seemingly paradoxical findings of the A allele being associated with (presumably) higher oxytocin and empathy, but also worse social outcomes, support the need for a more nuanced model regarding the role of oxytocin in modulating social-emotional behavior in humanss^23^. One interpretation of the *CD38* literature, as first suggested by Tabak et al.^19^, is that A carriers are more socially sensitive which, in the face of an interpersonal stressor, can make them vulnerable to heightened negative emotions. Such heightened negative emotions, in turn, could have undesirable consequences for relationships, especially if or when such negativity is in response to a relationship partner. As pointed out by Eisenberg et al.^24^, negative emotional arousal tends to engender a self-focus, and people who become especially anxious or distressed in response to another’s emotions often avoid dealing with the distressing situation as a way to cope with their own negative emotions. While the findings described above are consistent with this idea, to our knowledge, no study has directly assessed the relationship of *CD38* genotype with indicators of negative reactivity in an emotionally evocative situation. Moreover, many prior *CD38* studies rely on retrospective measures; however, it is well-known that such reconstructed memories can be influenced by various cognitive and motivational processes operating at the time of recall^25^. Assessing in-the-moment affective reactions can mitigate the effects of this potential recall bias and thus, arguably, capture an individual’s actual empathic responding tendencies with greater veracity.

To explore this possibility, we performed a secondary analysis of a dataset in which participants viewed an emotionally evocative video of a father discussing his child’s terminal cancer and rated their feelings of empathic concern (e.g., “sympathetic,” “tender,” “compassionate”) and personal distress (e.g., “anxious,” “frightened,” “disturbed”)^26^. In line with Tabak et al.’s^19^ theorizing, if *CD38* A allele carriers are more socially sensitive and emotionally reactive, we predicted they would report greater in-the-moment personal distress to the video than their CC counterparts. As participants also completed the Interpersonal Reactivity Index (IRI), a well-established measure of trait empathy with subscales for empathic concern and personal distress^27^, we were also able to test the association of *CD38* genotype with participants’ dispositional patterns of emotional responding.

## Results

### Group characteristics and questionnaire scores

Of the 173 participants in the original dataset^26^, the 171 participants for which both *CD38* genotype data and questionnaire scores were available are included in the present analysis. Participants were identified as having the AA (n = 24), AC (n = 77), or CC (n = 70) genotype. For the genetic analyses, in line with prior studies, participants were divided into AA/AC or CC genotype groups. Table 1 summarizes the demographic characteristics of the two groups. As the proportion of males and females differed between genotype groups, and sex differences in the emotion ratings are likely^28^, sex is included in the subsequent analyses.

**Table 1:** Summary of participant characteristics by CD38 rs3796863 genotype group.

		AA/AC	CC	*p*-value
	n	101	70	
	% Female	64%	49%	0.04*
	Age	20.1 + 3	20.5 + 1.8	0.26
Ethnicity	Caucasian	30%	24%	
	South Asian	39%	42.50%	
	East Asian	19%	24.50%	
	Other/Multiple	13%	9%	

*p < 0.05, independent sample *t*-test.

### Association of sex and CD38 genotype with emotional responses to the video

**Figure 1:**
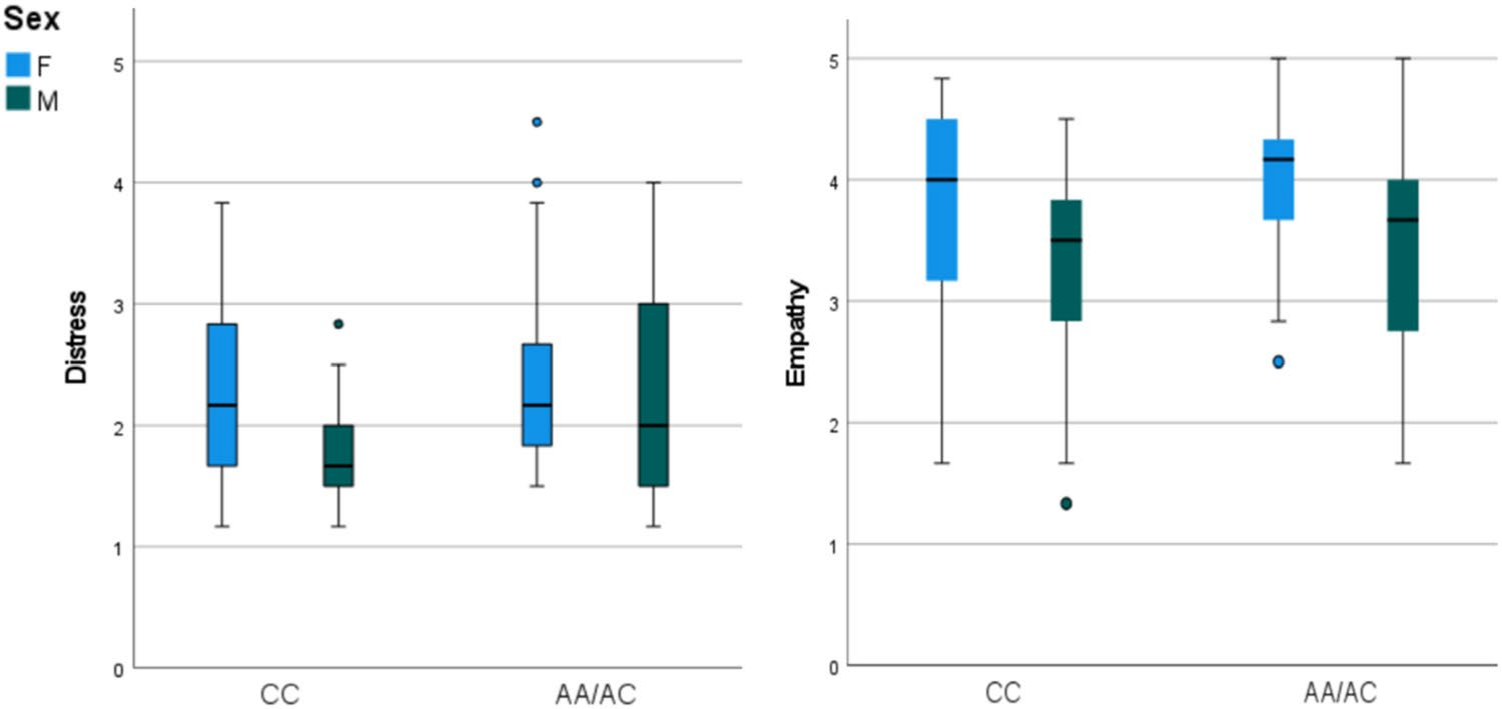
Boxplots comparing distress- and empathy-related responses to the video by sex and *CD38* genotype. For personal distress-related response, average scores were significantly higher for females and the AA/AC group. For empathy-related response, average scores were significantly higher for females.

Figure 1 shows the emotional responses to the video separated by sex and genotype. For distress-related response, there was a main effect of sex (F = 6.3, p = 0.01, η^2^ = 0.036) and *CD38* genotype (F = 4.6, p = 0.03, η^2^ = 0.027), but not their interaction (F = 2.5, p = 0.12, η^2^ = 0.015). The mean distress-related response ratings were higher for females than males (estimated marginal means and 95% confidence interval (95%CI): 2.3 [2.1–2.4] vs. 2.0 [1.8–2.2]) and AA/AC compared to CC genotype groups (2.3 [2.2–2.4] vs 2.1 [1.8–2.2]). For empathic concern-related response, only the main effect of sex was statistically significant (F = 21.2, p < 0.001, η^2^ = 0.11), with females scoring higher than males (3.9 [3.8–4.1] vs. 3.4 [3.2–3.5]). Empathic concern-related response scores were not significantly higher for the AA/AC group than the CC group (3.7 [3.6–3.9] vs. 3.5 [3.4–3.7]; F = 2.1, p = 0.15, η^2^ = 0.012).

### Association of sex and CD38 genotype with IRI subscales

To explore whether *CD38* genotype also related to dispositional measures of emotional responding, we examined the relationship with the personal distress and empathic concern subscales of the IRI. For both subscales, there was a significant main effect of sex with females scoring higher than males (personal distress: F = 4.1, p = 0.045, η^2^ = 0.024, mean [95% CI] = 13.7 [12.8–14.6] vs. 12.2 [11.2–13.3]; empathic concern: F = 16.9, p < 0.001, η^2^ = 0.092, 20.9 [20.0–21.9] vs. 17.8 [16.6–19.0]). Neither IRI subscale score was significantly higher for the AA/AC group than the CC group (personal distress: F = 3.1, p = 0.08, η^2^ = 0.018, mean [95% CI] = 13.6 [12.7–14.5] vs. 12.3 [11.2–13.4]; empathic concern: F = 1.9, p = 0.17, η^2^ = 0.011, mean [95% CI] = 19.9 [19.0–20.8] vs. 18.3 [17.7–20.0]).

## Discussion

Considerable research over the past two decades has highlighted the key role of oxytocin in human social-emotional processes. Growing evidence suggests that, rather than having broad prosocial effects, oxytocin may increase how attuned people are to social-emotional cues in their environment^23,29^ (for direct evidence of this in rodents, see^30,31^). This more nuanced mechanism better explains some of the disparate findings in the literature: if oxytocin augments the salience of social-emotional cues, then the effects of oxytocin should depend on the valence of those cues, facilitating prosocial behavior in positive, supportive social contexts^32^, but not in negative^33^ or uncertain^34^ contexts.

In line with this idea, Tabak et al.^19^ theorized that variation in *CD38* rs3796863—a putative oxytocin pathway gene^11,12^—may be associated with variation in social-emotional sensitivity, with A carriers being more socially sensitive than their CC counterparts; a similar hypothesis has been put forward for variation in the oxytocin receptor (*OXTR)* gene^35,36^. Like other “sensitivity genes”^37,38^, susceptibility to social-environmental influences influenced by *CD38* variation could lead to heightened emotional reactivity in threatening or emotionally distressing situations.

To our knowledge, the present study is the first to directly examine the effect of *CD38* rs3796863 variation on emotional reactivity. Consistent with our predictions, when confronted with another in distress, individuals with an A allele (vs. CC) reported significantly higher levels of personal distress, a self-focused, aversive emotional reaction. Conversely, the effect of genotype on feelings of empathic concern—an other-oriented response of care—was non-significant. While these two empathic responses are often simultaneously elicited in empathy-inducing situations and tend to be correlated^39,40^, they can have very different consequences for downstream helping behavior. Whereas empathic concern typically promotes helping behavior to alleviate the distress of the person in need, personal distress can induce a self-focused reaction aimed at alleviating one’s own distress that, consequently, can undermine helping behavior^41,42^. Such attunement to social-emotional cues may make *CD38* rs3796863 A allele carriers more accurate at detecting others’ emotions. Although there are no direct studies assessing the relationship between *CD38* and empathic accuracy, the finding that A allele carriers respond more sensitively to their children’s needs^14^ suggests higher empathic accuracy. While empathic accuracy (particularly for negative emotions) is associated with better relationship quality^43^, research indicates that greater empathic accuracy is also associated with heightened distress and anxiety during relationship conflict^44^^.^. Thus, a model that considers the context of the interpersonal interaction—namely whether the interaction is likely to evoke feelings of personal distress—can provide a parsimonious explanation for seeming discrepancies in the *CD38* literature. While *CD38* A allele carriers may express greater empathic responding when interacting with their own children in a supportive experimental setting^14^ or helping a hypothetical person through a financial donation^15^, they may also be more vulnerable to feelings of personal distress. To the extent that such feelings of personal distress engender a self-focus (which they often do), this self-focus could undermine social interactions and heighten perceptions of social disconnection, fueling negative mood ^18,19,21,22^. In line with the possibility that *CD38* rs3796863 influences susceptibility to negative mood in certain contexts, this SNP is also linked to higher neuroticism^45^.

In addition to the aforenoted effects on social-emotional sensitivity, it may be that *CD38* influences the ability to regulate anxiety and stress, given the link between *CD38* and oxytocin release^16^ and the well-established role of oxytocin in modulating anxiety and stress^46^ (see ^47^ for a similar hypothesis about oxytocin and emotion regulation). If stress regulation is impaired, individuals may feel more overwhelmed by their emotions—indeed, this is the essence of personal distress and what distinguishes personal distress from the more other-oriented response of empathic concern^24,48^. Future work should more systematically investigate the effects of *CD38* on emotion regulation, especially in interpersonal contexts.

Although we found an effect of *CD38* on state personal distress in response to the video stimulus, the relationship with the trait-like personal distress subscale of the IRI was not statistically significant (although the pattern and effect size estimate were similar). This difference may reflect a weakness of trait measures that should—but often do not—predict actual behavior^49^. We also found no association between *CD38* rs3796863 and the empathic concern subscale of the IRI, which is consistent with another study of 421 healthy individuals^50^. Trait measures based on subjective self-report assume that people have insight into, and can reliably report on, their own feelings and behavior; however, such insight is quite limited^51,52^. Moreover, people are often unaware of situational factors that can constrain their behavior^53^ and this bias can lead them to assign greater weight than is warranted to trait-based explanations, undermining the veracity of trait measures. These findings thus highlight the importance of using more naturalistic empathy paradigms when assessing emotional responding and the role of *CD38* therein.

If the oxytocin system indeed provides a buffer against stress and anxiety in response to challenging interpersonal situations, and oxytocin-related genetic variants can predict individuals for whom this buffer is weaker, genetic information may be useful for identifying individuals who are especially likely to struggle to regulate their negative emotions and thus most in need of support. In line with this possibility, Zeev-Wolf et al.^8^ found that a cumulative index of five oxytocin pathway genes (including *CD38* rs379686) predicted alterations in default mode network connectivity, which is thought to reflect anxiety and stress, among children exposed to chronic war-related trauma. Whether such individuals have greater likelihood of long-term negative interpersonal outcomes, and what interventions—whether pharmacological or psychological—may support them to cope with negative emotions in response to stress, will require longitudinal research.

While this study provides preliminary evidence that *CD38* influences distress-related responses to emotional stimuli, some limitations should be noted. First, given the relatively young age of the participants (university students), the video content involving a father describing his child’s terminal illness may not have been as emotionally salient as it would be for older adults, especially those who are parents. That said, the fact that we observed an effect here suggests that one might see even stronger *CD38* effects in a context that is more age- and life-stage-appropriate. Future studies should explore emotional responses to stimuli involving other interpersonal interactions, such as relationship conflict. Second, the functional significance of *CD38* variation in the human oxytocin system remains unclear and warrants further investigation. Based on the studies described in the Introduction^14,15^, we speculate that *CD38* A allele carriers experience higher oxytocin release in response to the stimulus, which in turn influences distress-related emotional responding. Finally, as our sample size is not large by current standards for genetic studies and focused on a single oxytocin-related genetic variant; future work is needed to replicate this pattern in a larger sample.

## Conclusion

The findings of this study provide preliminary support for the hypothesis that variation in the oxytocin pathway gene *CD38* influences social-emotional sensitivity, finding that A allele carriers are more prone to distress-related emotions in response to a negative social stressor. This hypothesis integrates the notion that stress reactivity can affect one’s emotional responding and may help reconcile the seemingly contradictory literature that *CD38* A allele carriers are more empathic (which was not found in the present study), yet have worse interpersonal outcomes. In contexts involving social conflict, even if empathy levels are high, high levels of personal distress may prevent appropriate social support from being provided. Overall, our findings suggest a more nuanced approach to understanding the relationship between oxytocin-related genetic variants and socially relevant outcomes and the need to consider in-the-moment emotional responses in addition to trait measures.

## Methods and materials

### Study design

This study is a secondary analysis of a dataset originally used to assess salivary hormone responses to an empathy-inducing video^26^. The three-minute video depicts a father narrating the story of his child’s terminal cancer and has been validated to induce emotional and hormonal responses^54^. Participants were recruited from a Canadian university and received course credit or payment ($10 CAD) for participation. The inclusion criteria were: (1) age 18 or older and (2) no health conditions expected to influence hormone levels. Participants were instructed not to eat, drink, smoke, chew gum, or brush their teeth for 1 h before providing the saliva samples. The study was approved by the Research Ethics Board of Simon Fraser University (study number 2015s0228) and conducted in accordance with the Declaration of Helsinki. All participants provided written informed consent.

### Ratings of emotional response to video

Immediately after watching the video, participants completed a questionnaire indicating on a scale from 1 to 5 how strongly they experienced 12 emotions (Supplementary Material). This questionnaire was adapted from the original study using the experimental video^54^ and is also based on Batson et al.’s^41^ state measures of empathic concern and personal distress. Of the 12 emotions, six assessed state feelings of empathic concern (*sympathetic, warm, compassionate, tender, soft-hearted,* and *moved*) and six assessed state feelings of personal distress (*anxious, annoyed, sad, distressed, frightened*, and *disturbed*). These six items were used to generate distress- and empathy-related video response scores ranging 1–5, with higher scores indicating greater endorsement of the emotional response.

### IRI subscales

The IRI was used to assess aspects of trait-like interpersonal emotional responding^27^. For the purpose of this secondary analysis, we focused on two subscales: (1) empathic concern, which measures “other-oriented feelings of sympathy and concern for unfortunate others”, and (2) personal distress, which measures “self-oriented personal anxiety and unease in tense interpersonal settings”. Responses were recorded on a 5-point Likert scale. Responses for the seven items per subscale were summed to generate a score of 0–28, with a higher score indicating increased levels of the trait. Participants completed the IRI approximately 10 min after watching the emotional video while waiting to provide a saliva sample.

### Genotyping

DNA was extracted from saliva samples using the standard phenol–chloroform method. *CD38* rs3796863 SNP genotyping was performed using a Roche LightCycler® 96 Real-Time PCR system. As this is a known variant, endpoint genotyping was performed using a TaqMan® SNP Genotyping Assay (C___1216944_10, which reports the complementary strand nucleotide). This method uses two differently labeled hydrolysis probes that bind to the target allele and release fluorescence at a specific wavelength. The resulting fluorescence data were analyzed using LightCycler® 96 software, version 1.1.0.1320, to automatically determine AA, AC, or CC genotypes.

In line with prior studies, statistical analyses compared outcomes between *CD38* rs3796863 A carrier (AA/AC) and CC genotype groups. Genotype frequencies did not deviate from Hardy–Weinberg equilibrium (p = 0.80) and are comparable to frequencies reported for other populations (e.g., 59% AA/AC and 41% CC for individuals of European ancestry living in Utah; see https://www.snpedia.com/index.php/Rs3796863 for genotype frequencies reported for various populations).

### Statistical analysis

Statistical analyses were performed using SPSS (IBM, USA). Multivariate analysis of variance with *CD38* genotype and sex as fixed effects, was used to explore the relationship of these variables with emotional response to the video (distress- and empathy-related) and IRI subscales (empathic concern and personal distress).

### Supplementary Information

Supplementary Information.

## Data Availability

The data analysed in this study are not publicly available because participants did not consent to having their genetic data deposited in a public repository, but are available from the corresponding author upon reasonable request.
