# Supplementary material for: CD38 genetic variation is associated with increased personal distress to an emotional stimulus
Source: Sci Rep. 2024 Jan 31;14:2571. doi: 10.1038/s41598-024-53081-5 (PMC10831108; doi:10.1038/s41598-024-53081-5)
Supplement: Supplementary file 1 — Supplementary Information. [file 41598_2024_53081_MOESM1_ESM.pdf]

Please refer to the word on the left, then circle the number that best represents how strongly you felt that emotion.

	**DID NOT FEEL THIS WAY AT ALL**				**STRONGLY FELT THIS WAY**
**1. Sympathetic**	1	2	3	4	5
**2. Warm**	1	2	3	4	5
**3. Anxious**	1	2	3	4	5
**4. Annoyed**	1	2	3	4	5
**5. Compassionate**	1	2	3	4	5
**6. Sad**	1	2	3	4	5
**7. Tender**	1	2	3	4	5
**8. Distressed**	1	2	3	4	5
**9. Soft-hearted**	1	2	3	4	5
**10. Frightened**	1	2	3	4	5
**11. Moved**	1	2	3	4	5
**12. Disturbed**	1	2	3	4	5
